# Barriers and facilitators to generating synthetic administrative data for research.

**DOI:** 10.23889/ijpds.v7i3.1984

**Published:** 2022-08-25

**Authors:** Theodora Kokosi, Bianca De Stavola, Robin Mitra, Andrew Copas, Katie Harron

**Affiliations:** 1 UCL GOS Institute of Child Health; 2 School of Mathematics, Cardiff University, Cardiff UK; 3 UCL Institute for Global Health, UK

## Objectives

Generation of synthetic data could improve the efficiency of administrative data analysis. We describe barriers and facilitators to synthetic administrative data in the UK based on our experience of generating, assessing, and evaluating the performance of different approaches. We aim to provide guidance on the appropriate uses of synthetic administrative data.

## Approach

We generated synthetic versions of one large-population survey (Natsal-3) and two administrative datasets (Hospital Episode Statistics [HES] and National Pupil Database [NPD]). A range of methods were used based on the statistical techniques of sampling and prediction. We implemented non-parametric (e.g., Classification and Regression Tree) and parametric (e.g., generalised linear models) methods, and multiple imputation and Bayesian networks in R software. We attempted to generate low- and high-fidelity datasets and assessed utility by visualising marginal distributions of key variables, estimating the standardised propensity mean square error, and deriving standardised coefficient differences of model estimates and overlap of confidence intervals.

## Results

Results from our analysis highlighted some facilitators related to low-fidelity synthetic data that are quicker to generate, can retain the data types, format, and privacy and could be used to support training and code development. Conversely, some of the barriers included computational issues when generating high-fidelity synthetic data from complex data structures. High-fidelity data are achievable but only in the context of a specific research question and a limited number of variables. Results from the Natsal-3 data showed that parametric methods produced slightly better data utility compared to non-parametric methods. Results for HES and NPD will also be presented.

## Conclusion

Low-fidelity synthetic data can provide a useful resource to support users of administrative data, whilst minimising data access timelines and while retaining privacy and confidentiality of personal data. High-utility datasets can be generated but take considerable resources, and current approaches cannot fully handle the complexity of longitudinal administrative data.

